# Bis[2-(ethoxy­carbonyl­amino)ethan­aminium] hexa­bromidostannate

**DOI:** 10.1107/S160053680904687X

**Published:** 2009-11-11

**Authors:** R. Alan Howie, Geraldo M. de Lima, Edward R. T. Tiekink, James L. Wardell, Solange M. S. V. Wardell

**Affiliations:** a Department of Chemistry, University of Aberdeen, Old Aberdeen AB15 5NY, Scotland; b Departamento de Quimica, ICEx, Universidade Federal de Minas Gerais, 31270-901 Belo Horizonte, MG, Brazil; c Department of Chemistry, University of Malaya, 50603 Kuala Lumpur, Malaysia; d CHEMSOL, 1 Harcourt Road, Aberdeen AB15 5NY, Scotland

## Abstract

In the title salt, (C_5_H_13_N_2_O_2_)_2_[SnBr_6_], the Sn atom (site symmetry 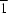) exists in a slightly distorted octa­hedral geometry. The cation is non-planar as the terminal CH_2_NH_3_^+^ residue lies below the plane defined by the remaining non-H atoms. In the crystal, cations associate *via* N—H⋯O hydrogen bonds involving the ammonium and carbonyl residues, forming a 14-membered {⋯HNC_2_NCO}_2_ synthon. The cations and anions are arranged in alternating layers arranged along the *a*-axis direction, the major association between them being N—H⋯Br contacts.

## Related literature

For background to the synthesis of the title salt, see: Duschinsky (1950 ▶); Kita *et al.* (1980 ▶); Smith *et al.* (1998 ▶); Tavridou *et al.* (1995 ▶); Wilson & Nowick (1998 ▶).
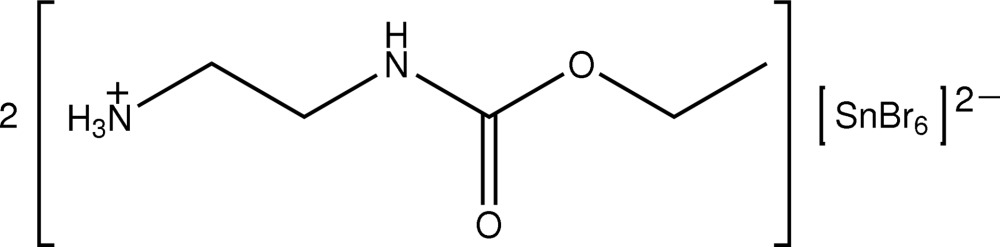


## Experimental

### 

#### Crystal data

(C_5_H_13_N_2_O_2_)_2_[SnBr_6_]
*M* *_r_* = 864.48
Monoclinic, 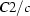

*a* = 21.8907 (5) Å
*b* = 7.4428 (2) Å
*c* = 15.5318 (4) Å
β = 105.934 (2)°
*V* = 2433.34 (11) Å^3^
*Z* = 4
Mo *K*α radiation
μ = 10.92 mm^−1^
*T* = 120 K
0.38 × 0.32 × 0.22 mm

#### Data collection

Bruker–Nonius 95mm CCD camera on κ-goniostat diffractometer
Absorption correction: multi-scan (*SADABS*; Sheldrick, 2003 ▶) *T*_min_ = 0.355, *T*_max_ = 0.746
15137 measured reflections
2777 independent reflections
2450 reflections with *I* > 2σ(*I*)
*R*_int_ = 0.051

#### Refinement

*R*[*F* ^2^ > 2σ(*F* ^2^)] = 0.034
*wR*(*F* ^2^) = 0.070
*S* = 1.12
2777 reflections
120 parameters
1 restraint
H-atom parameters constrained
Δρ_max_ = 0.87 e Å^−3^
Δρ_min_ = −1.35 e Å^−3^

### 

Data collection: *COLLECT* (Hooft, 1998 ▶); cell refinement: *DENZO* (Otwinowski & Minor, 1997 ▶) and *COLLECT*; data reduction: *DENZO* and *COLLECT*; program(s) used to solve structure: *SHELXS97* (Sheldrick, 2008 ▶); program(s) used to refine structure: *SHELXL97* (Sheldrick, 2008 ▶); molecular graphics: *DIAMOND* (Brandenburg, 2006 ▶); software used to prepare material for publication: *SHELXL97*.

## Supplementary Material

Crystal structure: contains datablocks global, I. DOI: 10.1107/S160053680904687X/hb5214sup1.cif

Structure factors: contains datablocks I. DOI: 10.1107/S160053680904687X/hb5214Isup2.hkl

Additional supplementary materials:  crystallographic information; 3D view; checkCIF report

## Figures and Tables

**Table 1:** Selected bond lengths (Å)

Sn—Br2	2.5820 (4)
Sn—Br3	2.6053 (4)
Sn—Br1	2.6075 (4)

**Table 2:** Hydrogen-bond geometry (Å, °)

*D*—H⋯*A*	*D*—H	H⋯*A*	*D*⋯*A*	*D*—H⋯*A*
N1—H1n⋯Br3^i^	0.88	2.83	3.501 (3)	134
N2—H2n⋯Br1^ii^	0.91	2.64	3.495 (3)	157
N2—H3n⋯Br3^iii^	0.91	2.84	3.425 (3)	123
N2—H4n⋯O1^iv^	0.91	1.88	2.717 (5)	152

Symmetry codes: (i) 
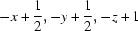
; (ii) 
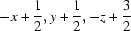
; (iii) 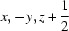; (iv) 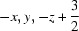.

**Fig. 1.**
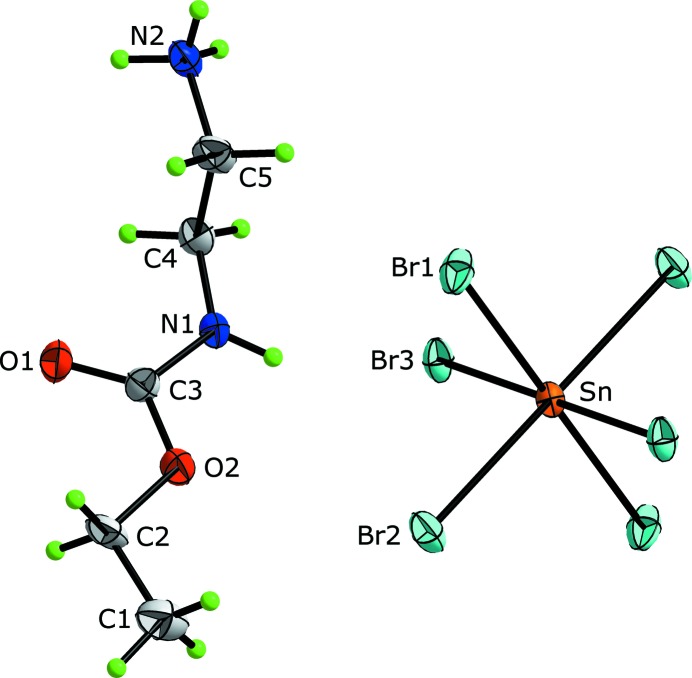
Molecular structure of (I) showing displacement ellipsoids at the 70% probability level. Unlabelled bromide ions are generated by the symmetry operation 1/2 - x, 1/2 - y, 1 - z.

**Fig. 2.**
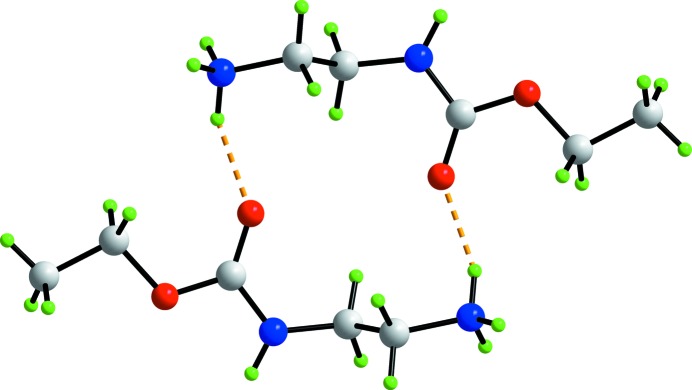
Supramolecular dimer in (I) mediated by N–H···O hydrogen bonds (orange dashed lines).

**Fig. 3.**
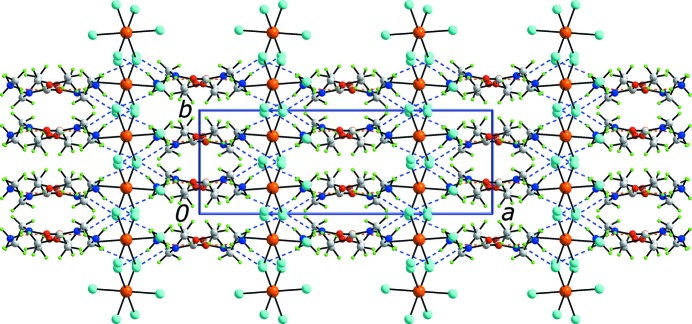
Unit-cell contents for (I) viewed in projection down the c axis. The N–H···O hydrogen bonds (orange dashed lines) and N–H···Br contacts (blue dashed lines) indicate the mode of asscoiated between the components of the crystal structure.

**Fig. 4.**
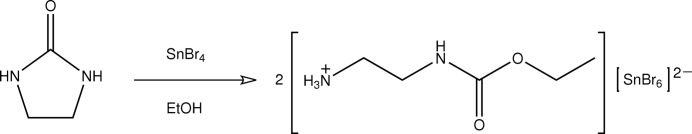
The formation of the title compound.

## supplementary crystallographic information

### Comment

Tin halides react with 2-imidazolidone, 1 (Scheme 2), in non-protic solvents such as dichloromethane, to form solid complexes [*R*_2_SnCl_2_(1-O)_2_] (*R* = Me, Bu or Ph), [MeSnCl_3_(1-O)_2_] and [Sn*X*_4_(1-O)_2_] (*X* = Cl, Br or I) (Tavridou *et al.*, 1995); 1-O is the O-bound form of 1. As reported herein, 2-imidazolidone reacts with SnBr_4_ in EtOH to form ethyl (2-ammonioethyl)carbamate hexabromostannate, (I), Fig. 4. The combination of SnBr_4_ and EtOH proved to have sufficient Brønsted acidity to open the 2-imidazidone ring. Ring opening reactions of 2-imidazolidone derivatives have been variously reported using bases, *e.g. N*-(2-nitrobenzenesulfonyl)-2-imidazolidon by a secondary amine, *R R*'NH (Wilson & Nowick, 1998) and acids, *e.g.*, ring opening of 1-methyl-3–3-hydroxyphenyl-2-imidaxolidone by concentrated HCl to give MeNHCH_2_CH_2_NHC_6_H_4_OH-m (Duschinsky, 1950). With reduced acidity, co-crystallization can occur instead as shown by the isolation of a 1:1 adduct of 2-imidazolidone and 5-nitrosalicylic acid (Smith *et al.*, 1998). The free base of 2 has been reported from the reaction of H_2_NCH_2_CH_2_NH_2_ with EtO_2_CO(MeO)CCH_2_ (Kita *et al.*, 1980).

The structure of (I) comprises ethyl (2-ammonioethyl)carbamate cations and SnBr_6_ dianions in the ratio 2:1; the Sn atom is located on a crystallographic centre of inversion so that the asymmetric unit is defined by one cation and half an anion, Fig. 1. The cation is not planar. While the RMS deviation of the O1, O2, N1 and C1—C4 atoms is 0.019 Å, the C5 atom lies 1.410 (5) Å out of this plane; the C3/N1/C4/C5 torsion angle of 96.4 (4)°. In the anion, the Sn—Br2 bond distance of 2.5820 (4) Å is significantly shorter than the Sn–Br1 and Sn–Br3 bond distances of 2.6075 (4) and 2.6053 (4) Å, respectively, an observation rationalized in terms of the pattern of intermolecular interactions,see below. The carbonyl-O1 atom forms a hydrogen bond with an ammonium-H atom to form a dimeric aggregate, Table 1 and Fig. 2. The resulting 14-membered {···HNC_2_NCO}_2_ synthon has twofold symmetry. The remaining acidic H atoms form contacts to the Br1 and Br3 atoms, Table 1, explaining the variation of the Sn–Br bond distances, whereby the Br2 atom not engaged in a significant intermolecular contact forms the shorter of the Sn–Br bonds. Globally, the crystal packing comprises alternating layers of cations and anions stacking along the a direction.

### Experimental

A solution of 2-imidazolidone (0.86 g) and SnBr_4_ (2.20 g) in EtOH (10 ml) was heated to 323 K for 15 minutes, cooled and maintained at room temperature to slowly form crystals of (I); m. pt. 459–463 K. IR(KBr) = 1692 cm^-1^.

### Refinement

All H atoms were located from a difference map but, were geometrically placed (C–H = 0.98–0.99 Å, and N–H = 0.88–0.91 Å) and refined as riding with *U*_iso_(H) = 1.2–1.5*U*_eq_(C, N). The maximum and minimum residual electron density peaks of 0.85 and 1.26 e Å^-3^, respectively, were located 1.81 Å and 0.82 Å from the S1 and Sn atoms, respectively.

### Crystal data

**Table:**

(C_5_H_13_N_2_O_2_)_2_[SnBr_6_]	*F*(000) = 1624
*M**_r_* = 864.48	*D*_x_ = 2.360 Mg m^−^^3^
Monoclinic, *C*2/*c*	Mo *K*α radiation, λ = 0.71069 Å
Hall symbol: -C 2yc	Cell parameters from 11507 reflections
*a* = 21.8907 (5) Å	θ = 2.9–27.5°
*b* = 7.4428 (2) Å	µ = 10.92 mm^−^^1^
*c* = 15.5318 (4) Å	*T* = 120 K
β = 105.934 (2)°	Block, light-yellow
*V* = 2433.34 (11) Å^3^	0.38 × 0.32 × 0.22 mm
*Z* = 4	

### Data collection

**Table:**

Bruker–Nonius 95mm CCD camera on κ-goniostat diffractometer	2777 independent reflections
Radiation source: Bruker-Nonius FR591 rotating anode	2450 reflections with *I* > 2σ(*I*)
graphite	*R*_int_ = 0.051
Detector resolution: 9.091 pixels mm^-1^	θ_max_ = 27.5°, θ_min_ = 3.1°
φ and ω scans	*h* = −28→28
Absorption correction: multi-scan (*SADABS*; Sheldrick, 2003)	*k* = −9→9
*T*_min_ = 0.355, *T*_max_ = 0.746	*l* = −20→20
15137 measured reflections	

### Refinement

**Table:**

Refinement on *F*^2^	Primary atom site location: structure-invariant direct methods
Least-squares matrix: full	Secondary atom site location: difference Fourier map
*R*[*F*^2^ > 2σ(*F*^2^)] = 0.034	Hydrogen site location: inferred from neighbouring sites
*wR*(*F*^2^) = 0.070	H-atom parameters constrained
*S* = 1.12	*w* = 1/[σ^2^(*F*_o_^2^) + (0.0263*P*)^2^ + 9.0406*P*] where *P* = (*F*_o_^2^ + 2*F*_c_^2^)/3
2777 reflections	(Δ/σ)_max_ = 0.001
120 parameters	Δρ_max_ = 0.87 e Å^−^^3^
1 restraint	Δρ_min_ = −1.35 e Å^−^^3^

### Special details

**Table:**

Geometry. All s.u.'s (except the s.u. in the dihedral angle between two l.s. planes) are estimated using the full covariance matrix. The cell s.u.'s are taken into account individually in the estimation of s.u.'s in distances, angles and torsion angles; correlations between s.u.'s in cell parameters are only used when they are defined by crystal symmetry. An approximate (isotropic) treatment of cell s.u.'s is used for estimating s.u.'s involving l.s. planes.
Refinement. Refinement of *F*^2^ against ALL reflections. The weighted *R*-factor *wR* and goodness of fit *S* are based on *F*^2^, conventional *R*-factors *R* are based on *F*, with *F* set to zero for negative *F*^2^. The threshold expression of *F*^2^ > 2σ(*F*^2^) is used only for calculating *R*-factors(gt) *etc*. and is not relevant to the choice of reflections for refinement. *R*-factors based on *F*^2^ are statistically about twice as large as those based on *F*, and *R*- factors based on ALL data will be even larger.

### Fractional atomic coordinates and isotropic or equivalent isotropic displacement parameters (Å^2^)

**Table:**

	*x*	*y*	*z*	*U*_iso_*/*U*_eq_	
Sn	0.2500	0.2500	0.5000	0.01268 (10)	
Br1	0.220893 (18)	0.02595 (5)	0.61162 (3)	0.02190 (11)	
Br2	0.135158 (17)	0.22636 (5)	0.39826 (3)	0.02122 (11)	
Br3	0.284421 (16)	−0.01941 (5)	0.41692 (3)	0.01712 (11)	
O1	−0.02161 (12)	0.3062 (4)	0.6285 (2)	0.0235 (6)	
O2	0.01221 (13)	0.2554 (4)	0.5051 (2)	0.0224 (6)	
N1	0.08208 (14)	0.3322 (5)	0.6316 (2)	0.0200 (7)	
H1N	0.1073	0.3259	0.5963	0.024*	
N2	0.14598 (16)	0.2592 (4)	0.8785 (2)	0.0183 (7)	
H2N	0.1749	0.3501	0.8905	0.027*	
H3N	0.1627	0.1606	0.9111	0.027*	
H4N	0.1103	0.2942	0.8931	0.027*	
C1	−0.0502 (2)	0.1802 (6)	0.3600 (3)	0.0277 (10)	
H1A	−0.0323	0.2857	0.3379	0.042*	
H1B	−0.0933	0.1584	0.3219	0.042*	
H1C	−0.0235	0.0751	0.3584	0.042*	
C2	−0.05232 (18)	0.2130 (6)	0.4539 (3)	0.0212 (9)	
H2A	−0.0812	0.3143	0.4561	0.025*	
H2B	−0.0677	0.1047	0.4785	0.025*	
C3	0.02071 (18)	0.2985 (5)	0.5912 (3)	0.0163 (8)	
C4	0.10453 (17)	0.3786 (5)	0.7254 (3)	0.0187 (8)	
H4A	0.0693	0.4315	0.7455	0.022*	
H4B	0.1387	0.4695	0.7339	0.022*	
C5	0.1296 (2)	0.2146 (6)	0.7810 (3)	0.0227 (9)	
H5A	0.0971	0.1185	0.7674	0.027*	
H5B	0.1679	0.1695	0.7657	0.027*	

### Atomic displacement parameters (Å^2^)

**Table:**

	*U*^11^	*U*^22^	*U*^33^	*U*^12^	*U*^13^	*U*^23^
Sn	0.01042 (17)	0.01213 (17)	0.0158 (2)	0.00012 (12)	0.00415 (14)	−0.00043 (13)
Br1	0.0233 (2)	0.01857 (19)	0.0286 (2)	0.00387 (15)	0.01518 (17)	0.00774 (16)
Br2	0.01230 (18)	0.0248 (2)	0.0236 (2)	0.00109 (15)	−0.00008 (16)	−0.00413 (16)
Br3	0.01406 (18)	0.01542 (18)	0.0230 (2)	−0.00038 (14)	0.00701 (15)	−0.00511 (15)
O1	0.0150 (13)	0.0372 (16)	0.0200 (16)	−0.0028 (12)	0.0075 (11)	−0.0051 (13)
O2	0.0141 (13)	0.0355 (17)	0.0170 (15)	0.0003 (11)	0.0034 (12)	−0.0037 (12)
N1	0.0116 (14)	0.0328 (19)	0.0161 (18)	−0.0009 (14)	0.0046 (13)	0.0002 (15)
N2	0.0125 (14)	0.0211 (16)	0.0195 (18)	0.0017 (12)	0.0016 (13)	−0.0030 (13)
C1	0.031 (2)	0.026 (2)	0.023 (2)	0.0041 (19)	0.0008 (18)	−0.0024 (18)
C2	0.0159 (18)	0.026 (2)	0.017 (2)	0.0009 (16)	−0.0038 (16)	−0.0016 (17)
C3	0.0164 (18)	0.0160 (17)	0.016 (2)	0.0001 (15)	0.0044 (15)	−0.0005 (15)
C4	0.0158 (17)	0.0199 (18)	0.019 (2)	−0.0042 (15)	0.0034 (15)	−0.0015 (16)
C5	0.0205 (19)	0.028 (2)	0.017 (2)	0.0045 (17)	−0.0002 (16)	−0.0079 (17)

### Geometric parameters (Å, °)

**Table:**

Sn—Br2^i^	2.5820 (4)	N2—H3N	0.9100
Sn—Br2	2.5820 (4)	N2—H4N	0.9100
Sn—Br3	2.6053 (4)	C1—C2	1.493 (6)
Sn—Br3^i^	2.6053 (4)	C1—H1A	0.9800
Sn—Br1^i^	2.6075 (4)	C1—H1B	0.9800
Sn—Br1	2.6075 (4)	C1—H1C	0.9800
O1—C3	1.222 (5)	C2—H2A	0.9900
O2—C3	1.339 (5)	C2—H2B	0.9900
O2—C2	1.453 (5)	C4—C5	1.509 (6)
N1—C3	1.341 (5)	C4—H4A	0.9900
N1—C4	1.445 (5)	C4—H4B	0.9900
N1—H1N	0.8800	C5—H5A	0.9900
N2—C5	1.494 (5)	C5—H5B	0.9900
N2—H2N	0.9100		
			
Br2^i^—Sn—Br2	180.000 (10)	C2—C1—H1B	109.5
Br2^i^—Sn—Br3	89.437 (12)	H1A—C1—H1B	109.5
Br2—Sn—Br3	90.563 (12)	C2—C1—H1C	109.5
Br2^i^—Sn—Br3^i^	90.563 (12)	H1A—C1—H1C	109.5
Br2—Sn—Br3^i^	89.437 (12)	H1B—C1—H1C	109.5
Br3—Sn—Br3^i^	180.0	O2—C2—C1	106.4 (3)
Br2^i^—Sn—Br1^i^	89.357 (13)	O2—C2—H2A	110.4
Br2—Sn—Br1^i^	90.643 (13)	C1—C2—H2A	110.4
Br3—Sn—Br1^i^	90.355 (12)	O2—C2—H2B	110.4
Br3^i^—Sn—Br1^i^	89.645 (12)	C1—C2—H2B	110.4
Br2^i^—Sn—Br1	90.643 (13)	H2A—C2—H2B	108.6
Br2—Sn—Br1	89.357 (13)	O1—C3—O2	124.8 (3)
Br3—Sn—Br1	89.646 (12)	O1—C3—N1	124.2 (4)
Br3^i^—Sn—Br1	90.354 (12)	O2—C3—N1	111.0 (3)
Br1^i^—Sn—Br1	180.000 (16)	N1—C4—C5	110.7 (3)
C3—O2—C2	116.5 (3)	N1—C4—H4A	109.5
C3—N1—C4	122.4 (3)	C5—C4—H4A	109.5
C3—N1—H1N	114.7	N1—C4—H4B	109.5
C4—N1—H1N	122.9	C5—C4—H4B	109.5
C5—N2—H2N	109.5	H4A—C4—H4B	108.1
C5—N2—H3N	109.5	N2—C5—C4	110.4 (3)
H2N—N2—H3N	109.5	N2—C5—H5A	109.6
C5—N2—H4N	109.5	C4—C5—H5A	109.6
H2N—N2—H4N	109.5	N2—C5—H5B	109.6
H3N—N2—H4N	109.5	C4—C5—H5B	109.6
C2—C1—H1A	109.5	H5A—C5—H5B	108.1
			
C3—O2—C2—C1	176.8 (3)	C4—N1—C3—O2	−178.8 (3)
C2—O2—C3—O1	−0.8 (6)	C3—N1—C4—C5	96.4 (4)
C2—O2—C3—N1	179.1 (3)	N1—C4—C5—N2	−173.7 (3)
C4—N1—C3—O1	1.1 (6)		

Symmetry codes: (i) −*x*+1/2, −*y*+1/2, −*z*+1.

### Hydrogen-bond geometry (Å, °)

**Table:**

*D*—H···*A*	*D*—H	H···*A*	*D*···*A*	*D*—H···*A*
N1—H1n···Br3^i^	0.88	2.83	3.501 (3)	134
N2—H2n···Br1^ii^	0.91	2.64	3.495 (3)	157
N2—H3n···Br3^iii^	0.91	2.84	3.425 (3)	123
N2—H4n···O1^iv^	0.91	1.88	2.717 (5)	152

Symmetry codes: (i) −*x*+1/2, −*y*+1/2, −*z*+1; (ii) −*x*+1/2, *y*+1/2, −*z*+3/2; (iii) *x*, −*y*, *z*+1/2; (iv) −*x*, *y*, −*z*+3/2.
